# Large Multidomain Protein NMR: HIV-1 Reverse Transcriptase Precursor in Solution

**DOI:** 10.3390/ijms21249545

**Published:** 2020-12-15

**Authors:** Tatiana V. Ilina, Zhaoyong Xi, Teresa Brosenitsch, Nicolas Sluis-Cremer, Rieko Ishima

**Affiliations:** 1 Department of Structural Biology, University of Pittsburgh School of Medicine, Pittsburgh, PA 15260, USA; tai4@pitt.edu (T.V.I.); zhx23@pitt.edu (Z.X.); tab24@pitt.edu (T.B.); 2 Department of Medicine, Division of Infectious Diseases, University of Pittsburgh School of Medicine, Pittsburgh, PA 15261, USA; nps2@pitt.edu

**Keywords:** HIV, reverse transcriptase, protein, NMR, ribonuclease H, isotope labeling

## Abstract

NMR studies of large proteins, over 100 kDa, in solution are technically challenging and, therefore, of considerable interest in the biophysics field. The challenge arises because the molecular tumbling of a protein in solution considerably slows as molecular mass increases, reducing the ability to detect resonances. In fact, the typical ^1^H-^13^C or ^1^H-^15^N correlation spectrum of a large protein, using a ^13^C- or ^15^N-uniformly labeled protein, shows severe line-broadening and signal overlap. Selective isotope labeling of methyl groups is a useful strategy to reduce these issues, however, the reduction in the number of signals that goes hand-in-hand with such a strategy is, in turn, disadvantageous for characterizing the overall features of the protein. When domain motion exists in large proteins, the domain motion differently affects backbone amide signals and methyl groups. Thus, the use of multiple NMR probes, such as ^1^H, ^19^F, ^13^C, and ^15^N, is ideal to gain overall structural or dynamical information for large proteins. We discuss the utility of observing different NMR nuclei when characterizing a large protein, namely, the 66 kDa multi-domain HIV-1 reverse transcriptase that forms a homodimer in solution. Importantly, we present a biophysical approach, complemented by biochemical assays, to understand not only the homodimer, p66/p66, but also the conformational changes that contribute to its maturation to a heterodimer, p66/p51, upon HIV-1 protease cleavage.

## 1. Introduction

Many retroviruses translate their proteins as large precursor polyproteins from which individual proteins are cleaved to become their mature functional forms [1]. Polyproteins are multi-domain proteins and are known to be mobile and adhesive to other proteins in solution, with complex folding and thermodynamic characteristics [2,3,4,5,6,7]. Thus, multiple orthogonal methods are essential to obtain a reliable characterization of such proteins in solution. In this review article, we focus on reverse transcriptase (RT) from human immunodeficiency virus-1 (HIV-1), which is initially expressed as a 66 kDa protein (p66) in a Gag-Pol polyprotein and contains five domains in itself. RT proteins play an essential role in the replication of all retroviruses, including HIV-1, and related retrotransposons [8,9,10,11,12].

During viral maturation, p66 is cleaved by the HIV-1 protease (PR) to form a functional heterodimer, comprising p66 and p51 subunits (p66/p51) [13,14]. The p51 subunit is generated upon PR-mediated removal of a majority of the ribonuclease H (RNH) domain from p66 [15,16,17]. Over the past ~25 years, a wealth of published structural data has elucidated the molecular details of the mature p66/p51 and its interactions with deoxyribonucleotide triphosphates and DNA substrates, as well as polymerase inhibitors and RNH inhibitors [18,19,20,21,22,23,24,25,26,27,28,29,30,31]. Generally, X-ray or NMR-based structure determination of a heterodimer, such as RT, is less straightforward than that of a symmetric homodimer because the number of constraints that are used in the structural calculation of a homodimer is half that of a heterodimer. However, in the case of RT, structural characterization begins with the heterodimer. 

As we describe below, one reason for this is the susceptibility of p66/p66 to protease digestion, relative to p66/p51 which is a more protease-protected form. Indeed, when HIV-1 researchers first began in vitro production of p66/p51 RT, the protein was prepared in *E. coli*, by encoding only the 66 kDa protein, without the HIV-1 protease, because the *E. coli* enzymes could process the p66 subunit to p51 [21,32,33]. In addition to its greater susceptibility to proteases, p66/p66 has a weaker dimer affinity compared to p66/p51, with an approximate dissociation constant of 4 µM compared to 0.3 µM, respectively [34,35,36]. With the advancement of recombinant protein expression and purification, studies to structurally characterize p66/p66 have become possible during the past several years. Remarkably, the structure of the immature p66/p66 homodimer remains unknown even though its structure is expected to provide important information for RT maturation [37,38,39,40]. We will highlight how the 132 kDa dimeric protein has been studied by solution NMR and how other biophysics/biochemistry methods have been used to verify or validate the results.

## 2. Function, Structure, and Maturation of HIV-1 RT 

### 2.1. Function and Structure of the Mature HIV-1 RT

RT catalyzes all steps in the reverse transcription of the HIV-1 (+) single stranded RNA into double stranded DNA and is, therefore, essential for virus replication [14,41,42,43]. It has been a primary target for antiviral drug development since the discovery of HIV-1 in 1983, and 12 agents that directly target this enzyme have been FDA approved as HIV-1 antivirals (plus several more in clinical trials) [44,45,46]. These antivirals can be categorized into two therapeutic classes: nucleoside/nucleotide RT inhibitors (NRTIs) and nonnucleoside RT inhibitors (NNRTIs). Both NRTIs and NNRTIs bind in (NRTIs) or near to (NNRTIs) the DNA polymerase domain of RT, and primarily impact this activity [47,48]. However, RT is a multifunctional enzyme and also contains an RNH domain that is responsible for the cleavage of the RNA strand in the intermediate RNA/DNA duplex that is formed during reverse transcription [13,14]. To date, despite significant effort [49,50,51,52,53,54,55,56,57], no drug that targets this function has been clinically developed.

**Figure 1:**
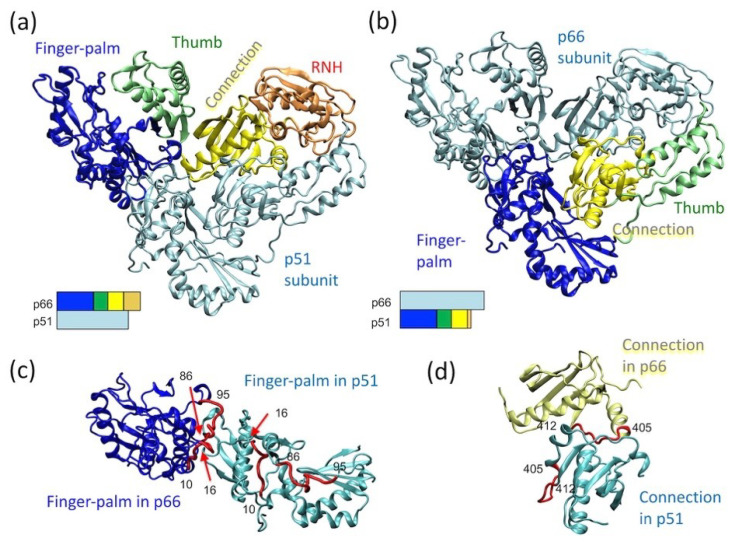
p66/p51 reverse transcriptase (RT) structure, highlighting (**a**) the domain orientation in the p66 subunit, (**b**) the domain orientation in the p51 subunit, and relative orientation of (**c**) two finger–palm domains in the p66 and p51 subunits and that of (**d**) the two connection domains in the p66 and p51 subunits. In panels (**a**,**b**), the bar presentations below the structures indicate which domains are highlighted: finger–palm (blue), thumb (green), connection (yellow) and ribonuclease H (RNH) (orange). In panel (**c**), residues, 10–16 and 86–95, that are at the subunit interface in the p66 subunit, are highlighted with a red color in both subunits. Similarly, in panel (**d**), residues, 405–412, that are at the subunit interface in the p66 subunit, are highlighted in both subunits. The graphic presentation was made using VMD software [65] and the RT structure (PDB 1DLO [66]).

The p66 subunit in mature RT, p66/p51, has two domains: a polymerase domain (residues 1 to 426) and an RNH domain (residues 427 to 560). The polymerase domain contains finger–palm (residues 1 to 236), thumb (residues 237 to 318), and connection (residues 319 to 426) subdomains, while the RNH domain is a single domain fold (residues 427 to 560) (blue, green, yellow, orange ribbons, respectively, in Figure 1a) [20]. Among published reports, the term “subdomain” is not always utilized [58,59,60,61] and, the starting or ending residue numbers may differ slightly, based on differences in the allocation of a β-strand or a loop region [18,19,62,63]. Although the finger–palm subdomain has two structurally distinct regions, i.e., finger (residues 1 to 85 and 120 to 150) and palm (residues 85 to 119 and 151 to 243), they are not sequentially independent of each other, and thus are combined as finger–palm in this review article. In RT, the polymerase active site, D110, D185, and D186, is located in the finger–palm domain [64] while the ribonuclease active site, D443, E478, D498, and D549, is located in the RNH domain. 

The p51 subunit in p66/p51 lacks the RNH domain, and the relative orientation of the finger–palm, thumb, and connection domains differ in the p51 subunit compared to the p66 (Figure 1b). Although the finger–palm and connection domains in the p66 subunit interact with those in the p51 subunit, the domains in the p66 subunit do not arrange symmetrically with those in the p51 subunit (Figure 1c,d). For example, the subunit interface residues of the finger–palm domain of p66 are located at the inner core of the finger–palm domain in the p51 subunit (Figure 1c). Similarly, a part of the subunit interface residues in the connection domain in the p66 subunit interact with the finger–palm in the p51 subunit (Figure 1d). The conformational rearrangements leading to this p66/p51 conformation is structurally of interest and is discussed further below.

**Figure 2:**
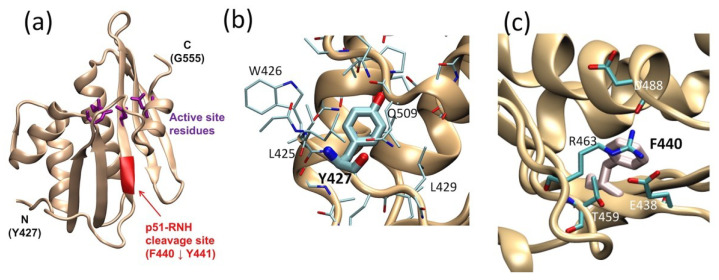
RNH domain structure, highlighting (**a**) the p51-RNH processing site (i.e., F440–Y441, red ribbon) and the active site (purple sticks), (**b**) the Y427 side chain (thick stick), and (**c**) F440 cleavage site. In panels (**b**,**c**) the side chains of the surrounding residues are shown. The graphic presentation was made using VMD software [65] and the RT structures (PDB 3KK2 [72] or 1DLO [66]).

For RT maturation, HIV-1 PR cleaves one of the p66 subunits in p66/p66 at residues 440 and 441; the location of this cleavage site, often referred to as the “p51-RNH processing site”, is significant since the RNH domain in p66 begins at residue 427 (Figure 2a). As such, the processing site, F440–Y441, is actually within the RNH fold and not in the domain-linker. This is clearly evident in the RNH domain structure of the full-length heterodimer and in the structure of the isolated RNH protein: the terminal regions are exposed to solution while the p51-RNH cleavage site of p66 is sequestered in a β-sheet within the RNH domain and is inaccessible to PR (Figure 2a) [18,19,44,67]. Previous backbone dynamic studies of an isolated RNH domain showed that residues F440–Y441 are rigidly folded in the protein core in solution [68,69]. In the mature protein, p66/p51, this cleavage site is also protected by the side chain of Y427; although the stretch of amino acids from Y427 to T439 appears to have no secondary structure elements, it tightly interacts with the RNH core, with the side chain of Y427 capping a pocket of the RNH core (Figure 2b) [70]. Evidence for a stabilizing effect of this region on the core comes from studies showing that E438N or F440A mutation unfolds the RNH core (Figure 2c) [71]. Further, molecular dynamics (MD) simulation of the wild type and mutant RNH indicate the importance of the charge network at the processing site (Figure 2c) [71]. Although the F440 cleavage site residue must be exposed to solution for PR access, its side chain is also needed to maintain the RNH core fold. Thus, in p66/p51, the p51-RNH processing site is protected, and to understand the structural basis of RT maturation, an understanding of the precursor, p66/p66, structure is essential.

### 2.2. Maturation Mechanism of HIV-1 RT

HIV-1 encodes three polyproteins: Gag, Gag-Pol, and Env. HIV-1 Env contains gp41 and gp120, and Env complex formation and trafficking have been studied [73,74,75,76]. HIV-1 Gag polyprotein encodes the matrix, capsid, and nucleocapsid proteins along with the p6 peptide; the structural properties of the Gag maturation intermediates, from the precursor form to functional forms, have been extensively characterized [77,78,79,80,81,82,83,84,85,86,87,88,89]. The Gag-Pol polyprotein encodes three enzymes, PR, RT, and IN, in addition to the proteins encoded in Gag. In contrast to the studies on HIV-1 Gag polyprotein, the versatile nature and maturation process of Gag-Pol are less studied.

Gag-Pol proteins are processed to matured proteins in both the cell and virus, however, the proteins found in the virus are matured in the virus [90,91,92,93,94]. Evidence for the latter comes from studies showing the accumulation of full-length Gag-Pol in the virus when the protein is packaged to the virus in the presence of a PR inhibitor or with an inactive PR mutant [90,91,92,93]. Further, when RT and/or IN are deleted from Gag-Pol, a delivery vehicle (i.e., a Gag-derived packaging signal or sequence) is required for their packaging into the virus (the enzymes alone are not packaged) [95]. 

The first event of Gag-Pol precursor processing is a cleavage at the p2/NC site in Gag by a cis (intra-molecular) PR reaction, while subsequent processing steps of the Pol region involve a trans (inter-molecular) reaction [77,81,82,83,96]. In experiments on cells or viruses, p66 is produced before, or near the same time as, p51 [37,92,97]. (Note, the processing order observed in in vitro transcription/translation experiments [81,82,96] is different from those observed with in vivo systems, presumably because of differences in the protein dimerization and oligomerization steps as well as molecular interactions with cellular factors). Importantly, mutations in the RT region are known to affect Gag-Pol polyprotein maturation in the virion [92,98], suggesting the importance of the p66 region for the maturation of the entire Pol protein. Although the sequence of PR processing at sites within the Pol region differ among reports, mutation studies and protein concentration studies consistently indicate that p66 homodimer formation is a prerequisite for p66/p51 formation [37,40,99]. 

**Figure 3:**
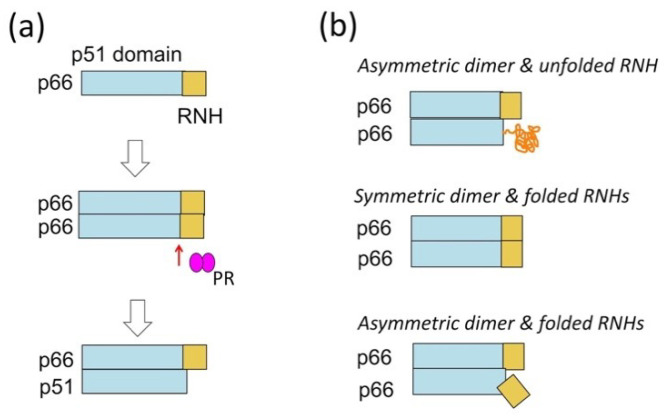
(**a**) RT maturation process, i.e., the formation of p66/p66 and processing to p66/p51, and (**b**) three homodimer structural models: asymmetric homodimer with one unfolded RNH domain, proposed based on solution NMR experiments of p66/p66, p66/p51, and p51 [70,101,102]; symmetric homodimer with both RNH domains folded, proposed based on solution NMR experiments of p66/p66 and p51 [100]; or asymmetric homodimer with both RNH domains folded, proposed based on electron spin resonance (ESR) experiments [103].

These observations indicate that the immediate upstream step to p66/p51 RT formation is formation of p66/p66 and cleavage of the homodimer to p66/p51 by PR (Figure 3a). The dissociation constant (K_D_) of p66/p66, ~4 µM, is ten times larger than that of p66/p51 [34,35,36,100], indicating that tight dimerization occurs after p51-RNH cleavage, and this dimerization and associated conformational change likely prevent the cleavage of the remaining RNH domain in p66/p51. However, the structure of p66/p66 and the solution exposure of the uncleaved p51-RNH site are unknown. We and others have tried to characterize the p66/p66 structure, providing three structural models: asymmetric homodimer with one unfolded RNH domain, based on solution NMR experiments of p66/p66, p66/p51, and p51 [70,101,102]; symmetric homodimer with both RNH domains folded, based on solution NMR experiments of p66/p66 and p51 [100]; or asymmetric homodimer with both RNH domains folded, based on electron spin resonance (ESR) experiments [103] (Figure 3b). 

While NMR data of a protein in solution reflects the ensemble average of conformers of the protein in a certain time scale, ESR data provide the ensemble of conformers as a set of snap shots of the conformers, regardless of the time scale, by flash freezing. Thus, the two folded RNH models, whether conformational symmetry is present or not, may be consistent with each other. On the other hand, RNH unfolding requires an energy barrier to be crossed, because the folded form is protected, as discussed in the previous subsection (Figure 2). We will discuss the model differences in the last section of this review.

## 3. NMR and Other Methods to Understand Conformational Characteristics of p66/p66 

### 3.1. NMR Strategy to Observe p66/p66

Solution NMR studies of large proteins, over 100 kDa, are technically challenging and of considerable interest in the NMR field [104,105,106]. The slowing of molecular tumbling in solution as molecular mass increases accounts for this challenge; severe line-broadening and signal overlap occur in the typical ^1^H-^13^C or ^1^H-^15^N correlation spectra obtained from large proteins uniformly labeled with ^13^C- or ^15^N. Selective isotope labeling, often concomitant with deuteration, is well-known as a strategy to reduce this signal overlap and line-broadening [107]. In addition, transverse relaxation optimized spectroscopy (TROSY)-based experiments increase the upper limit of molecular size that can be studied in solution by NMR [108,109,110,111]. In particular, methyl groups are highly sensitive NMR probes and are often used to detect NMR signals of large proteins in solution [105,112,113,114,115,116]. This is because the fast three-site jump of methyl groups significantly reduces the line-width of the methyl NMR signals, making them easier to detect compared to signals with broad line-widths [117,118,119,120] and establishing the equivalency of the three C-H vectors that undergo dipolar cross correlation, naturally enabling the TROSY effect in the HMQC experiments [110,111]. 

Taking advantage of the methyl ^1^H and ^13^C signals, the London group pioneered efforts to assess the conformational differences in p51 RT, p66 RT, and p66/p51 RT by observing methionine methyl signals in the HMQC experiments [121,122]. They initially identified the effect of NNRTI interaction on the p66 subunit and the p51 subunit of p66/p51 RT. Proteins needed for the Met-ε methyl NMR can be prepared by expressing the protein in a minimum medium or a defined amino-acid medium with ^13^C-ε labeled methionine. This Met-ε methyl NMR is sensitive and has been applied to investigate various NNRTI bound forms of p66/p51 by other groups, too [123,124]. 

The London group next applied methyl NMR using RTs that were perdeuterated but with ^1^H/^13^C labeling at the isoleucine δ1 positions, mainly to understand the p66/p66 structure and maturation. Based on differences in their data at the start versus the end of a many hour experiment, they derived a model in which p66/p66 forms a symmetric conformation but undergoes a slow conformational change, with a 6.5 h time constant, to an asymmetric form, in which one RNH domain is unfolded (“Asymmetric dimer & unfolded RNH” model in Figure 2b) [63,70,101,102,125]. In both methionine methyl and isoleucine methyl studies, they integrated NMR results with their small angle X-ray scattering (SAXS) data and/or MD simulation results [63,70,101,102,125]. 

**Figure 4:**
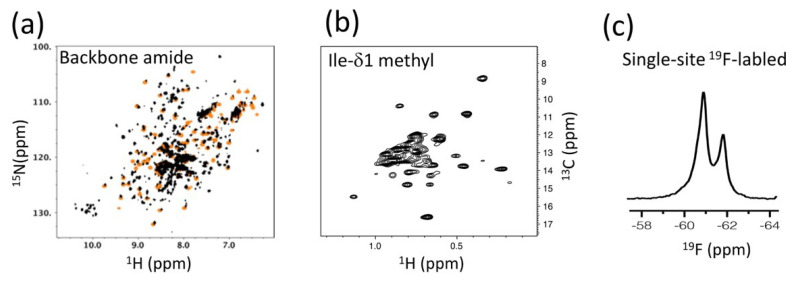
NMR strategies used to investigate RT: (**a**) an example of a ^1^H-^15^N TROSY HSQC spectrum, used to compare p66 with those of isolated domains; (**b**) an example of an Ile-δ1 methyl ^1^H-^13^C HMQC spectrum, used for comparison of the different states of p66/p66; and (**c**) a one-dimensional ^19^F NMR spectrum of a single-site labeled p66/p66. In panel (**a**), HSQC signals from an isolated RNH (orange) are overlaid as an example.

Our efforts to understand the p66/p66 homodimer conformation in solution began with a comparison of ^1^H-^15^N NMR spectra from the p66 protein (residues from 1 to 560) with those from the isolated thumb domain (residues from 237 to 318), the RNH domain (residues from 427 to 560), and p51 (residues from 1 to 427) (Figure 4a) [100]. Generally, comparison of NMR spectra from isolated domains with those of an entire protein is a useful and practical strategy to study large proteins that undergo domain motion [105,126,127]. Nevertheless, it was impossible to detect all signals of the 132 kDa homodimer protein by using ^1^H-^15^N TROSY-HSQC. In fact, we were surprised by the many signals that were observed in the p66 NMR spectrum [100]. The p66 spectral pattern was similar to that of p51, which homodimerizes with a K_D_ of ~0.3 mM, compared to the approximate ten-fold weaker K_D_ of p66/p66 [34,35,36,100]. Superimposing the p66 and p51 spectra with those of isolated thumb and RNH domains indicated a high degree of similarity among the spectra [100]. Dimerization of p66, >80%, was confirmed from size-exclusion chromatography with multi-angle light scattering (SEC-MALS). Given that only one set of resonances appeared to be present in the p66 spectrum, we assumed that the p66 monomer-dimer equilibrium affects fast-exchange on the chemical shift time scale. On this assumption, the similarity of the spectral patterns among the isolated domains, p51 and p66, indicated independent domain motions of the thumb and RNH domains in p66/p66. We proposed a “symmetric dimer & folded RNHs” model of the p66/p66 conformation, based on these observations (Figure 2b).

The limited sensitivity of the ^1^H-^15^N NMR experiments prompted us to follow-up using the strategy of the London group, i.e., detection of Ile-δ1 methyl signals of p66/p66 (Figure 4b) [128]. We recorded ^1^H-^13^C HMQC spectra of Ile-δ1 methyl signals of p66/p66 in the inhibitor-free form, the NNRTI-bound form, and those with/without tRNA^Lys3^ [128], a known primer for the reverse transcription reaction that, with the viral RNA, interacts with RT [129,130,131]. In the absence of PR, we observed a stable p66/p66 spectrum that did not change over a period of 40 h [128]. Particularly note-worthy was the absence of a time-dependent increase in the random coil signal of the Ile-δ1 methyl spectrum. Even upon titration of an NNRTI that enhances homodimer formation (described below), the random coil signal was not increased and only slight shifts of methyl signals in the RNH domain were observed, suggesting that the RNH domains were folded in the apo- and NNRTI-bound forms [128]. In the presence of tRNA^Lys3^, either with or without NNRTI, one RNH domain has an altered conformation or experiences a different chemical environment. RT maturation experiments in vitro (i.e., monitoring the proteolysis of p66/p66 to p66/p51 by PR) showed that p66/p66 in the presence of tRNA^Lys3^, either with/without NNRTI, is more efficiently matured [128], correlating the states that were observed by NMR with the professing efficiency in the states. We also utilized information obtained from our ^19^F NMR data regarding the position of 181 in p66/p66 and p66/p51 in the absence or presence of NNRTI (Figure 4c) [132,133]. The data suggested a 1:1 NNRTI:p66/p66 binding stoichiometry and conformational similarity between p66/p66 and p66/p51 at residue 181.

Finally, we compared ^1^H-^13^C HMQC spectra of Ile-δ1 methyl signals of p66/p66 in the various ligand-bound states [128] and correlated these with the efficiency of p66/p51 production in the presence of active PR using similar conditions [40], with an aim to identify what conformation enhances RT maturation. The production of p66/p51 by PR was enhanced in the presence of tRNA^Lys3^, regardless of whether NNRTI was present. As mentioned above, significant chemical shift changes of one RNH domain were also observed in the presence of tRNA^Lys3^, regardless of NNRTI binding. Generally, the slow PR-catalyzed production of p66/p51 in the absence of tRNA^Lys3^ is consistent with the conclusion that both RNH domains are folded in the unbound homodimer. Altogether, these data consistently support a “symmetric dimer & folded RNHs” model (Figure 2b). The significance of the p66/p66 structure models is discussed below. 

### 3.2. Characteristics of Methyl vs Amide NMR When Domain Motion Exists

The Ile-δ1 and Met-ε methyl HMQC experiment, as discussed thus far, is highly sensitive and, therefore, informative when characterizing large proteins in solution [105,112,113,114,115,116,121,122], due to the fast three-site jump of methyl groups [117,118,119,120], and the TROSY effect, which is a consequence of the methyl group C-H dipolar cross-correlations [110,111]. Ile-δ1 methyl HMQC signals have been observed both in rigid and mobile regions of large proteins. In contrast, our initial ^1^H-^15^N HSQC experiments, in which many ^1^H-^15^N cross peaks were observed, suggested more domain motion [70,100], than our Ile-δ1 methyl HMQC spectra. Although the intrinsic sensitivity of ^1^H-^13^C methyl-TROSY and ^1^H-^15^N TROSY experiments differ, the effect of domain motion upon effective correlation times is expected to be similar for the two experiments. 

**Figure 5:**
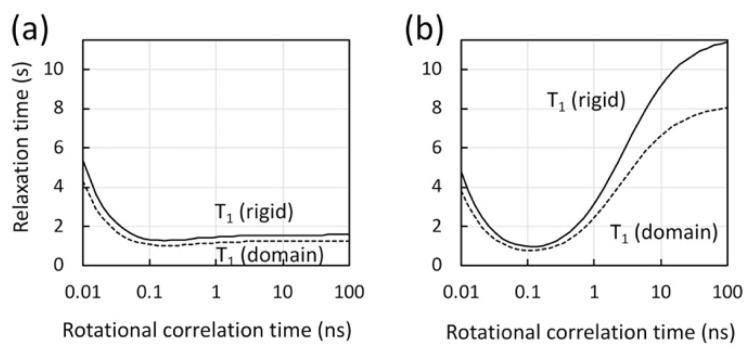
Calculated longitudinal relaxation time (T_1_) for protons in methyl geometry, (**a**) with and (**b**) without methyl fast rotation, assuming a rigid molecule (solid line) and a molecule with a domain motion at 4 ns (dashed line), as a function of the molecular rotational correlation time at 900 MHz. In the calculations, a model-free spectral density function with an extended fast motion was assumed [140,141] with a generalized order parameter (S_s_^2^) of 0.8, and with a correlation time for internal motion of 50 ps for a rigid molecule (solid lines) or 4 ns for one that undergoes domain motion (dashed lines). In the calculation in panel (**a**), an order parameter for methyl fast motion (S_f_^2^) was assumed at 0.25 for methyl protons [117,139]. In the calculation in panel (**b**), S_f_^2^ at 0.9 was assumed to provide a relaxation sink.

Here, we discuss three possibilities that could cause differences in the “apparent sensitivity” to domain motion in ^1^H-^13^C methyl-TROSY and ^1^H-^15^N TROSY experiments. First, Ile-δ1 methyl groups experience more rotational internal dynamics than the backbone amide [134] and, thus, are expected to be less sensitive to domain motion or overall molecular tumbling, when compared to the backbone amides. Second, since the ^1^H spin–flip rate increases as the rotational correlation time increases, domain motion may significantly decrease ^1^H spin–flips among the residual ^1^Hs in a perdeuterated protein and, thus, enhance ^1^H-^15^N TROSY effects [135,136]. Third, magnetization recovery during the pulse repetition time may also affect the overall spectral sensitivity in the spectra. To discuss the latter point, we show plots of the proton longitudinal relaxation time (T_1_) with methyl fast rotation (Figure 5a) and without methyl fast rotation (Figure 5b). The latter mimics the case of amide proton T_1_, yet we did not directly compare this result with the calculated amide proton relaxation rates in deuterated proteins because the latter T_1_’s vary greatly, depending on the level of deuteration and individual sites [137,138]. 

The rotational correlation time is estimated at ~100 ns for the p66/p66 homodimer (the right edge of the graph, in Figure 5). Our calculation illuminates that the proton magnetization recovery is significantly shorter for methyl protons that undergo fast methyl rotation, compared to non-methyl protons (Figure 5) whose rotational correlation time is that of the protein. For proteins that have a rotational correlation time larger than ~ns, methyl ^1^H T_1_ is almost independent of the rotational correlation time of the protein, because of the fast methyl three-site jump [119,120,139] (Figure 5a). The calculation also shows that, although the T_1_ of non-methyl protons at ~100 ns rotational correlation time is large, it is significantly reduced when there is domain motion (Figure 5b). Taken together, there are multiple factors that affect the apparent sensitivity changes by domain motion in ^1^H-^13^C methyl-TROSY and ^1^H-^15^N TROSY experiments.

### 3.3. Integration of Multiple NMR Strategies

In addition to ^1^H-^15^N TROSY-HSQC and Ile-δ1 methyl HMQC, we also utilized ^19^F NMR data of a ^19^F single-site labeled protein to gain information in a pin-point manner. In these ^19^F NMR experiments, a ^19^F NMR probe, 4-trifluoromethyl-phenylalanine (tfmF), was site-specifically introduced using an orthogonal tRNA/tRNA synthetase pair [132,142,143]. Generally, the combination of a single-site label that prevents resonance overlap and the excellent sensitivity of ^19^F NMR (83% of ^1^H) enables the detection of changes in the chemical or structural environment at a specific site in a large protein [132,133]. Such an application of ^19^F NMR is selective, in that one can choose the specific location of the label, and is orthogonal to other NMR methods. The latter is important to validate observations from ^1^H-^15^N TROSY-HSQC and Ile-δ1 methyl HMQC NMR. Similarly, in the London group’s pioneering RT NMR work, multiple methods to validate observations were utilized, including methionine methyl NMR [121,122] and Ile-δ1 methyl NMR [70,101,102], as well as ^1^H-^15^N NMR [125]. Together, these studies demonstrate the importance of multi-nuclear NMR approaches, as well as other methodologies, which we further describe below. 

### 3.4. NMR and Other Methodologies

As initially mentioned, the “asymmetric dimer & unfolded RNH” model of p66/p66 was proposed by the London group [63,70,101,102,125], while we proposed the “symmetric dimer & folded RNHs” model [100,128] (Figure 3b). Since both models are based on NMR observations, a consideration of other model-validating methodologies is important to understand what was actually observed. 

**Figure 6:**
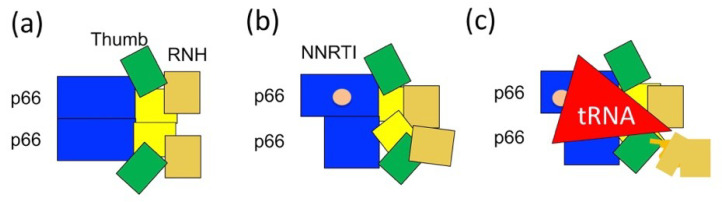
Our current model for p66/p51 production from p66/p66: (**a**) symmetric p66/p66 conformation in which thumb and RNH domains undergo domain motion; (**b**) asymmetric p66/p66 bound to NNRTI; and (**c**) conformational change induced by tRNA^Lys3^ interaction, regardless of the presence of NNRTI or not [40,100,124]. Note the degrees of conformational asymmetry in (**b**,**c**) are hypothetical.

In our case, we compared conformational states observed by NMR [128] with the efficiency of RT maturation by PR [40]. Production of p66/p51 by PR was slow in both the presence and absence of NNRTI, compared to those in the presence of tRNA^Lys3^ [40]. Such a difference in RT maturation rate is consistent with the structural model in which the RNH domains are folded in the absence of nucleic acid (Figure 6a) and even in the presence of NNRTI (Figure 6b). Upon tRNA^Lys3^ interaction, the p66/p66 NMR chemical shifts broadened or changed position for one of the two RNH domains [128]. Since our processing assay indicated that tRNA^Lys3^ enhances RT maturation [40], we concluded that conformation of p66/p66 in complex with tRNA^Lys3^ is a form in which one RNH domain is efficiently cleaved by PR (Figure 6c). In addition, we made sure that our p66 did not contain nucleic acid contamination based on the 254/280 ratio [40], or protease contamination, conducting SDS-PAGE on the NMR samples before and after the experiments to validate the species that were observed [128]. 

The London group’s MODEL was highly supported by their NMR data and other structural biology data, such as SAXS and MD simulations. The data explained the mechanistic structural changes as p66/p66 conformation became a p66/p51-like form and likely crossed a high-energy barrier from a folded RNH to unfolded RNH [63,70,101,102,125]. No assay to investigate actual p66/p51 production or biochemical characterization of the samples was shown in their studies. Since our group and the London group used an almost identical p66 amino acid sequence, the different observations may be explained by a difference in the relative domain orientations of the p66/p66 proteins, possibly introduced at the initial folding step during protein production or due to minor cofactors present in the samples (or not). In particular, we wonder whether their p66/p66 would mature to p66/p51 or p66/p5Xs, in which p5Xs is a molecular size between 66 kDa and 51 kDa. In addition, if the RNH is fully unfolded as suggested by their model, then intermediate products with lengths between those of p51 and p66, as well as p51 must be produced upon PR-mediated processing. Indeed, in a high-salt condition without tRNA^Lys3^, we observed PR-proteolyzed products in the SDS-PAGE between the p51 and p66 bands [40]. However, intermediate species are not detected in the virus [37,90,98,144]. Thus, we favor a model where the RNH domains in p66/p66 are folded, and one RNH is destabilized to enable specific cleavage at the p51-RNH site. Alternatively, structural studies of the Pol region, in addition to isolated p66, may further answer the RT maturation mechanism question.

## 4. Conclusions

This article reviewed recent work on the structural characterization of the large, multi-domain HIV-1 RT precursor, p66/p66, by application of multiple NMR probes and other biochemical and biophysical methodologies. We argue that inconsistent observations can be solved using an orthogonal approach. Given that multiple conformers may co-exist in multi-domain proteins, we conclude that such integrated methodologies are critical to gain insight into the validity of the models generated.

## Abbreviations

HIV-1	Human immunodeficiency virus-1
RT	Reverse transcriptase
RNH	Ribonuclease H
PR	Protease
NMR	Nuclear magnetic resonance
NRTI	Nucleoside/nucleotide RT inhibitor
NNRTI	Nonnucleoside RT inhibitor
SAXS	Small angle X-ray scattering
SEC	Size-exclusion chromatography
MALS	Multi-angle light scattering
tfmF	4-trifluoromethyl-phenylalanine
LysRS	Lysyl-tRNA synthetase

## References

[1] Yost S.A., Marcotrigiano J. (2013). Viral precursor polyproteins: Keys of regulation from replication to maturation. Curr. Opin. Virol..

[2] Batey S., Scott K.A., Clarke J. (2006). Complex folding kinetics of a multidomain protein. Biophys J..

[3] Pawson T. (2007). Dynamic control of signaling by modular adaptor proteins. Curr. Opin. Cell Biol..

[4] Koglin A., Walsh C.T. (2009). Structural insights into nonribosomal peptide enzymatic assembly lines. Nat. Prod. Rep..

[5] Walsh J.D., Meier K., Ishima R., Gronenborn A.M. (2010). NMR studies on domain diffusion and alignment in modular GB1 repeats. Biophys. J..

[6] Brosey C.A., Chagot M.E., Chazin W.J. (2012). Preparation of the modular multi-domain protein RPA for study by NMR spectroscopy. Methods Mol. Biol.

[7] Thompson M.K., Ehlinger A.C., Chazin W.J. (2017). Analysis of Functional Dynamics of Modular Multidomain Proteins by SAXS and NMR. Methods Enzymol..

[8] Baltimore D. (1970). RNA-dependent DNA polymerase in virions of RNA tumour viruses. Nature.

[9] Temin H.M., Mizutani S. (1970). RNA-dependent DNA polymerase in virions of Rous sarcoma virus. Nature.

[10] Temin H.M. (1993). Retrovirus variation and reverse transcription: Abnormal strand transfers result in retrovirus genetic variation. Proc. Natl. Acad. Sci. USA.

[11] Wilhelm M., Wilhelm F.X. (2001). Reverse transcription of retroviruses and LTR retrotransposons. Cell. Mol. Life Sci..

[12] Eickbush T.H., Jamburuthugoda V.K. (2008). The diversity of retrotransposons and the properties of their reverse transcriptases. Virus Res..

[13] Katz R.A., Skalka A.M. (1994). The retroviral enzymes. Annu. Rev. Biochem..

[14] Coffin J.M., Hughes S.H., Varmus H.E. (1997). Retroviruses.

[15] Chattopadhyay D., Evans D.B., Deibel M.R., Vosters A.F., Eckenrode F.M., Einspahr H.M., Hui J.O., Tomasselli A.G., Zurcher-Neely H.A., Heinrikson R.L. (1992). Purification and characterization of heterodimeric human immunodeficiency virus type 1 (HIV-1) reverse transcriptase produced by in vitro processing of p66 with recombinant HIV-1 protease. J. Biol. Chem..

[16] Sharma S.K., Fan N., Evans D.B. (1994). Human immunodeficiency virus type 1 (HIV-1) recombinant reverse transcriptase. Asymmetry in p66 subunits of the p66/p66 homodimer. FEBS Lett..

[17] Divita G., Rittinger K., Geourjon C., Deleage G., Goody R.S. (1995). Dimerization kinetics of HIV-1 and HIV-2 reverse transcriptase: A two step process. J. Mol. Biol..

[18] Kohlstaedt L.A., Wang J., Friedman J.M., Rice P.A., Steitz T.A. (1992). Crystal structure at 3.5 A resolution of HIV-1 reverse transcriptase complexed with an inhibitor. Science.

[19] Jacobo-Molina A., Ding J., Nanni R.G., Clark A.D.J., Lu X., Tantillo C., Williams R.L., Kamer G., Ferris A.L., Clark P. (1993). Crystal structure of human immunodeficiency virus type 1 reverse transcriptase complexed with double-stranded DNA at 3.0 A resolution shows bent DNA. Proc. Natl. Acad. Sci. USA.

[20] Wang J., Smerdon S.J., Jager J., Kohlstaedt L.A., Rice P.A., Friedman J.M., Steitz T.A. (1994). Structural basis of asymmetry in the human immunodeficiency virus type 1 reverse transcriptase heterodimer. Proc. Natl. Acad. Sci. USA.

[21] Clark A.D., JacoboMolina A., Clark P., Hughes S.H., Arnold E. (1995). Crystallization of human immunodeficiency virus type 1 reverse transcriptase with and without nucleic acid substrates, inhibitors, and an antibody fab fragment. Methods Enzymol..

[22] Ren J., Esnouf R., Garman E., Somers D., Ross C., Kirby I., Keeling J., Darby G., Jones Y., Stuart D. (1995). High resolution structures of HIV-1 RT from four RT-inhibitor complexes. Nat. Struct Biol..

[23] Jaeger J., Restle T., Steitz T.A. (1998). The structure of HIV-1 reverse transcriptase complexed with an RNA pseudoknot inhibitor. EMBO J..

[24] Ren J., Nichols C., Bird L.E., Fujiwara T., Sugimoto H., Stuart D.I., Stammers D.K. (2000). Binding of the second generation non-nucleoside inhibitor S-1153 to HIV-1 reverse transcriptase involves extensive main chain hydrogen bonding. J. Biol. Chem..

[25] Pata J.D., Stirtan W.G., Goldstein S.W., Steitz T.A. (2004). Structure of HIV-1 reverse transcriptase bound to an inhibitor active against mutant reverse transcriptases resistant to other nonnucleoside inhibitors. Proc. Natl. Acad. Sci. USA.

[26] Ren J., Nichols C.E., Chamberlain P.P., Weaver K.L., Short S.A., Stammers D.K. (2004). Crystal structures of HIV-1 reverse transcriptases mutated at Codons 100, 106 and 108 and mechanisms of resistance to non-nucleoside inhibitors. J. Mol. Biol..

[27] Morningstar M.L., Roth T., Farnsworth D.W., Smith M.K., Watson K., Buckheit R.W., Das K., Zhang W.Y., Arnold E., Julias J.G. (2007). Synthesis, biological activity, and crystal structure of potent nonnucleoside inhibitors of HIV-1 reverse transcriptase that retain activity against mutant forms of the enzyme. J. Med. Chem..

[28] Das K., Sarafianos S.G., Clark A.D., Boyer P.L., Hughes S.H., Arnold E. (2007). Crystal structures of clinically relevant Lys103Asn/Tyr181Cys double mutant HIV-1 reverse transcriptase in complexes with ATP and non-nucleoside inhibitor HBY 097. J. Mol. Biol..

[29] Das K., Bauman J.D., Clark A.D., Frenkel Y.V., Lewi P.J., Shatkin A.J., Hughes S.H., Arnold E. (2008). High-resolution structures of HIV-1 reverse transcriptase/TMC278 complexes: Strategic flexibility explains potency against resistance mutations. Proc. Natl. Acad. Sci. USA.

[30] Tu X., Das K., Han Q., Bauman J.D., Clark A.D., Hou X., Frenkel Y.V., Gaffney B.L., Jones R.A., Boyer P.L. (2010). Structural basis of HIV-1 resistance to AZT by excision. Nat. Struct Mol. Biol..

[31] Wright D.W., Sadiq S.K., De Fabritiis G., Coveney P.V. (2012). Thumbs Down for HIV: Domain Level Rearrangements Do Occur in the NNRTI-Bound HIV-1 Reverse Transcriptase. J. Am. Chem. Soc..

[32] D’Aquila R.T., Summers W.C. (1988). HIV-1 reverse transcriptase/ribonuclease H: High level expression in Escherichia coli from a plasmid constructed using the polymerase chain reaction. J. Acquir. Immune Defic. Syndr..

[33] Jacobo-Molina A., Clark A.D., Williams R.L., Nanni R.G., Clark P., Ferris A.L., Hughes S.H., Arnold E. (1991). Crystals of a ternary complex of human immunodeficiency virus type 1 reverse transcriptase with a monoclonal antibody Fab fragment and double-stranded DNA diffract X-rays to 3.5-A resolution. Proc. Natl. Acad. Sci. USA.

[34] Restle T., Muller B., Goody R.S. (1990). Dimerization of human immunodeficiency virus type 1 reverse transcriptase. A target for chemotherapeutic intervention. J. Biol. Chem..

[35] Sluis-Cremer N., Dmitrienko G.I., Balzarini J., Camarasa M.J., Parniak M.A. (2000). Human immunodeficiency virus type 1 reverse transcriptase dimer destabilization by 1-{spiro[4″-amino-2″,2″-dioxo-1″,2″-oxathiole-″,3′-[2′,5′-bis-O-(tert-butyldimethylsilyl)-beta-D-ribofuranosyl]]}-3-ethylthymine. Biochemistry.

[36] Venezia C.F., Howard K.J., Ignatov M.E., Holladay L.A., Barkley M.D. (2006). Effects of efavirenz binding on the subunit equilibria of HIV-1 reverse transcriptase. Biochemistry.

[37] Sluis-Cremer N., Arion D., Abram M.E., Parniak M.A. (2004). Proteolytic processing of an HIV-1 pol polyprotein precursor: Insights into the mechanism of reverse transcriptase p66/p51 heterodimer formation. Int. J. Biochem. Cell Biol..

[38] Abram M.E., Parniak M.A. (2005). Virion instability of human immunodeficiency virus type 1 reverse transcriptase (RT) mutated in the protease cleavage site between RT p51 and the RT RNase H domain. J. Virol..

[39] Abram M.E., Sarafianos S.G., Parniak M.A. (2010). The mutation T477A in HIV-1 reverse transcriptase (RT) restores normal proteolytic processing of RT in virus with Gag-Pol mutated in the p51-RNH cleavage site. Retrovirology.

[40] Ilina T.V., Slack R.L., Elder J.H., Sarafianos S.G., Parniak M.A., Ishima R. (2018). Effect of tRNA on the Maturation of HIV-1 Reverse Transcriptase. J. Mol. Biol..

[41] Gilboa E., Mitra S.W., Goff S., Baltimore D. (1979). A detailed model of reverse transcription and tests of crucial aspects. Cell.

[42] Hu W.S., Hughes S.H. (2012). HIV-1 reverse transcription. Cold Spring Harb. Perspect. Med..

[43] Le Grice S.F. (2012). Human immunodeficiency virus reverse transcriptase: 25 years of research, drug discovery, and promise. J. Biol. Chem..

[44] Jacobo-Molina A., Arnold E. (1991). HIV reverse transcriptase structure-function relationships. Biochemistry.

[45] Arnold E., Das K., Ding J., Yadav P.N., Hsiou Y., Boyer P.L., Hughes S.H. (1996). Targeting HIV reverse transcriptase for anti-AIDS drug design: Structural and biological considerations for chemotherapeutic strategies. Drug Des. Discov..

[46] Erickson J.W., Burt S.K. (1996). Structural mechanisms of HIV drug resistance. Annu. Rev. Pharmacol. Toxicol..

[47] Jochmans D. (2008). Novel HIV-1 reverse transcriptase inhibitors. Virus Res..

[48] De Clercq E. (2013). The nucleoside reverse transcriptase inhibitors, nonnucleoside reverse transcriptase inhibitors, and protease inhibitors in the treatment of HIV infections (AIDS). Adv. Pharmacol..

[49] Borkow G., Fletcher R.S., Barnard J., Arion D., Motakis D., Dmitrienko G.I., Parniak M.A. (1997). Inhibition of the ribonuclease H and DNA polymerase activities of HIV-1 reverse transcriptase by N-(4-tert-butylbenzoyl)-2-hydroxy-1-naphthaldehyde hydrazone. Biochemistry.

[50] Shaw-Reid C.A., Munshi V., Graham P., Wolfe A., Witmer M., Danzeisen R., Olsen D.B., Carroll S.S., Embrey M., Wai J.S. (2003). Inhibition of HIV-1 ribonuclease H by a novel diketo acid, 4-[5-(benzoylamino)thien-2-yl]-2,4-dioxobutanoic acid. J. Biol. Chem..

[51] Budihas S.R., Gorshkova I., Gaidamakov S., Wamiru A., Bona M.K., Parniak M.A., Crouch R.J., McMahon J.B., Beutler J.A., Le Grice S.F. (2005). Selective inhibition of HIV-1 reverse transcriptase-associated ribonuclease H activity by hydroxylated tropolones. Nucleic. Acids Res..

[52] Takada K., Bermingham A., O’Keefe B.R., Wamiru A., Beutler J.A., Le Grice S.F., Lloyd J., Gustafson K.R., McMahon J.B. (2007). An HIV RNase H inhibitory 1,3,4,5-tetragalloylapiitol from the African plant Hylodendron gabunensis. J. Nat. Prod..

[53] Bokesch H.R., Wamiru A., Le Grice S.F., Beutler J.A., McKee T.C., McMahon J.B. (2008). HIV-1 ribonuclease H inhibitory phenolic glycosides from Eugenia hyemalis. J. Nat. Prod..

[54] Sluis-Cremer N., Tachedjian G. (2008). Mechanisms of inhibition of HIV replication by non-nucleoside reverse transcriptase inhibitors. Virus Res..

[55] Kirschberg T.A., Balakrishnan M., Squires N.H., Barnes T., Brendza K.M., Chen X., Eisenberg E.J., Jin W., Kutty N., Leavitt S. (2009). RNase H active site inhibitors of human immunodeficiency virus type 1 reverse transcriptase: Design, biochemical activity, and structural information. J. Med. Chem..

[56] Chung S., Wendeler M., Rausch J.W., Beilhartz G., Gotte M., O’Keefe B.R., Bermingham A., Beutler J.A., Liu S., Zhuang X. (2010). Structure-activity analysis of vinylogous urea inhibitors of human immunodeficiency virus-encoded ribonuclease H. Antimicrob. Agents Chemother..

[57] Tang J., Vernekar S.K.V., Chen Y.L., Miller L., Huber A.D., Myshakina N., Sarafianos S.G., Parniak M.A., Wang Z. (2017). Synthesis, biological evaluation and molecular modeling of 2-Hydroxyisoquinoline-1,3-dione analogues as inhibitors of HIV reverse transcriptase associated ribonuclease H and polymerase. Eur. J. Med. Chem..

[58] Julias J.G., McWilliams M.J., Sarafianos S.G., Alvord W.G., Arnold E., Hughes S.H. (2003). Mutation of amino acids in the connection domain of human immunodeficiency virus type 1 reverse transcriptase that contact the template-primer affects RNase H activity. J. Virol..

[59] Cen S., Niu M., Kleiman L. (2004). The connection domain in reverse transcriptase facilitates the in vivo annealing of tRNALys3 to HIV-1 genomic RNA. Retrovirology.

[60] Yap S.H., Sheen C.W., Fahey J., Zanin M., Tyssen D., Lima V.D., Wynhoven B., Kuiper M., Sluis-Cremer N., Harrigan P.R. (2007). N348I in the connection domain of HIV-1 reverse transcriptase confers zidovudine and nevirapine resistance. PLoS Med..

[61] Nikolenko G.N., Delviks-Frankenberry K.A., Palmer S., Maldarelli F., Fivash M.J., Coffin J.M., Pathak V.K. (2007). Mutations in the connection domain of HIV-1 reverse transcriptase increase 3′-azido-3′-deoxythymidine resistance. Proc. Natl. Acad. Sci. USA.

[62] Seckler J.M., Howard K.J., Barkley M.D., Wintrode P.L. (2009). Solution structural dynamics of HIV-1 reverse transcriptase heterodimer. Biochemistry.

[63] London R.E. (2019). HIV-1 Reverse Transcriptase: A Metamorphic Protein with Three Stable States. Structure.

[64] Singh K., Marchand B., Kirby K.A., Michailidis E., Sarafianos S.G. (2010). Structural Aspects of Drug Resistance and Inhibition of HIV-1 Reverse Transcriptase. Viruses.

[65] Humphrey W., Dalke A., Schulten K. (1996). VMD: Visual molecular dynamics. J. Mol. Graph..

[66] Hsiou Y., Ding J., Das K., Clark A.D.J., Hughes S.H., Arnold E. (1996). Structure of unliganded HIV-1 reverse transcriptase at 2.7 A resolution: Implications of conformational changes for polymerization and inhibition mechanisms. Structure.

[67] Davies J.F., Hostomska Z., Hostomsky Z., Jordan S.R., Matthews D.A. (1991). Crystal structure of the ribonuclease H domain of HIV-1 reverse transcriptase. Science.

[68] Powers R., Clore G.M., Stahl S.J., Wingfield P.T., Gronenborn A.M. (1992). Analysis of the backbone dynamics of the ribonuclease H domain of the human immunodeficiency virus reverse transcriptase using nitrogen-15 relaxation measurements. Biochemistry.

[69] Mueller G.A., Pari K., DeRose E.F., Kirby T.W., London R.E. (2004). Backbone dynamics of the RNase H domain of HIV-1 reverse transcriptase. Biochemistry.

[70] Zheng X.H., Pedersen L.C., Gabel S.A., Mueller G.A., Cuneo M.J., DeRose E.F., Krahn J.M., London R.E. (2014). Selective unfolding of one Ribonuclease H domain of HIV reverse transcriptase is linked to homodimer formation. Nucleic Acids Res..

[71] Slack R.L., Spiriti J., Ahn J., Parniak M.A., Zuckerman D.M., Ishima R. (2015). Structural integrity of the ribonuclease H domain in HIV-1 reverse transcriptase. Proteins.

[72] Lansdon E.B., Samuel D., Lagpacan L., Brendza K.M., White K.L., Hung M., Liu X., Boojamra C.G., Mackman R.L., Cihlar T. (2010). Visualizing the molecular interactions of a nucleotide analog, GS-9148, with HIV-1 reverse transcriptase-DNA complex. J. Mol. Biol..

[73] McCune J.M., Rabin L.B., Feinberg M.B., Lieberman M., Kosek J.C., Reyes G.R., Weissman I.L. (1988). Endoproteolytic cleavage of gp160 is required for the activation of human immunodeficiency virus. Cell.

[74] Freed E.O., Myers D.J., Risser R. (1989). Mutational analysis of the cleavage sequence of the human immunodeficiency virus type 1 envelope glycoprotein precursor gp160. J. Virol..

[75] Hallenberger S., Bosch V., Angliker H., Shaw E., Klenk H.D., Garten W. (1992). Inhibition of furin-mediated cleavage activation of HIV-1 glycoprotein gp160. Nature.

[76] Checkley M.A., Luttge B.G., Freed E.O. (2011). HIV-1 envelope glycoprotein biosynthesis, trafficking, and incorporation. J. Mol. Biol..

[77] Wiegers K., Rutter G., Kottler H., Tessmer U., Hohenberg H., Krausslich H.G. (1998). Sequential steps in human immunodeficiency virus particle maturation revealed by alterations of individual Gag polyprotein cleavage sites. J. Virol..

[78] Ganser B.K., Li S., Klishko V.Y., Finch J.T., Sundquist W.I. (1999). Assembly and analysis of conical models for the HIV-1 core. Science.

[79] Briggs J.A., Simon M.N., Gross I., Krausslich H.G., Fuller S.D., Vogt V.M., Johnson M.C. (2004). The stoichiometry of Gag protein in HIV-1. Nat. Struct Mol. Biol..

[80] Pettit S.C., Gulnik S., Everitt L., Kaplan A.H. (2003). The dimer interfaces of protease and extra-protease domains influence the activation of protease and the specificity of GagPol cleavage. J. Virol..

[81] Pettit S.C., Everitt L.E., Choudhury S., Dunn B.M., Kaplan A.H. (2004). Initial cleavage of the human immunodeficiency virus type 1 GagPol precursor by its activated protease occurs by an intramolecular mechanism. J. Virol..

[82] Pettit S.C., Lindquist J.N., Kaplan A.H., Swanstrom R. (2005). Processing sites in the human immunodeficiency virus type 1 (HIV-1) Gag-Pro-Pol precursor are cleaved by the viral protease at different rates. Retrovirology.

[83] Lee S.K., Potempa M., Swanstrom R. (2012). The choreography of HIV-1 proteolytic processing and virion assembly. J. Biol. Chem..

[84] Byeon I.J.L., Meng X., Jung J.W., Zhao G.P., Yang R.F., Ahn J.W., Shi J., Concel J., Aiken C., Zhang P.J. (2009). Structural Convergence between Cryo-EM and NMR Reveals Intersubunit Interactions Critical for HIV-1 Capsid Function. Cell.

[85] Jouvenet N., Simon S.M., Bieniasz P.D. (2009). Imaging the interaction of HIV-1 genomes and Gag during assembly of individual viral particles. Proc. Natl. Acad. Sci. USA.

[86] Kutluay S.B., Bieniasz P.D. (2010). Analysis of the initiating events in HIV-1 particle assembly and genome packaging. PLoS Pathog..

[87] Sundquist W.I., Krausslich H.G. (2012). HIV-1 assembly, budding, and maturation. Cold Spring Harb. Perspect. Med..

[88] Deshmukh L., Ghirlando R., Clore G.M. (2014). Investigation of the structure and dynamics of the capsid-spacer peptide 1-nucleocapsid fragment of the HIV-1 gag polyprotein by solution NMR spectroscopy. Angew. Chem. Int. Ed. Engl..

[89] Deshmukh L., Ghirlando R., Clore G.M. (2015). Conformation and dynamics of the Gag polyprotein of the human immunodeficiency virus 1 studied by NMR spectroscopy. Proc. Natl. Acad. Sci. USA.

[90] Shehu-Xhilaga M., Crowe S.M., Mak J. (2001). Maintenance of the Gag/Gag-Pol ratio is important for human immunodeficiency virus type 1 RNA dimerization and viral infectivity. J. Virol..

[91] Hill M., Tachedjian G., Mak J. (2005). The packaging and maturation of the HIV-1 Pol proteins. Curr. HIV Res..

[92] Chiang C.C., Wang S.M., Tseng Y.T., Huang K.J., Wang C.T. (2009). Mutations at human immunodeficiency virus type 1 reverse transcriptase tryptophan repeat motif attenuate the inhibitory effect of efavirenz on virus production. Virology.

[93] Mattei S., Anders M., Konvalinka J., Krausslich H.G., Briggs J.A., Muller B. (2014). Induced maturation of human immunodeficiency virus. J. Virol..

[94] Freed E.O. (2015). HIV-1 assembly, release and maturation. Nat. Rev. Microbiol..

[95] Wu X., Liu H., Xiao H., Conway J.A., Hunter E., Kappes J.C. (1997). Functional RT and IN incorporated into HIV-1 particles independently of the Gag/Pol precursor protein. EMBO J..

[96] Pettit S.C., Clemente J.C., Jeung J.A., Dunn B.M., Kaplan A.H. (2005). Ordered processing of the human immunodeficiency virus type 1 GagPol precursor is influenced by the context of the embedded viral protease. J. Virol..

[97] Speck R.R., Flexner C., Tian C.J., Fu X.F. (2000). Comparison of human immunodeficiency virus type 1 Pr55(Gag) and Pr160(Gag-Pol) processing intermediates that accumulate in primary and transformed cells treated with peptidic and nonpeptidic protease inhibitors. Antimicrob. Agents Chemother..

[98] Dunn L.L., McWilliams M.J., Das K., Arnold E., Hughes S.H. (2009). Mutations in the thumb allow human immunodeficiency virus type 1 reverse transcriptase to be cleaved by protease in virions. J. Virol..

[99] Sudo S., Haraguchi H., Hirai Y., Gatanaga H., Sakuragi J., Momose F., Morikawa Y. (2013). Efavirenz Enhances HIV-1 Gag Processing at the Plasma Membrane through Gag-Pol Dimerization. J. Virol..

[100] Sharaf N.G., Poliner E., Slack R.L., Christen M.T., Byeon I.J., Parniak M.A., Gronenborn A.M., Ishima R. (2014). The p66 immature precursor of HIV-1 reverse transcriptase. Proteins.

[101] Zheng X., Perera L., Mueller G.A., DeRose E.F., London R.E. (2015). Asymmetric conformational maturation of HIV-1 reverse transcriptase. Elife.

[102] Zheng X.H., Pedersen L.C., Gabel S.A., Mueller G.A., DeRose E.F., London R.E. (2016). Unfolding the HIV-1 reverse transcriptase RNase H domain—How to lose a molecular tug-of-war. Nucleic. Acids Res..

[103] Schmidt T., Schwieters C.D., Clore G.M. (2019). Spatial domain organization in the HIV-1 reverse transcriptase p66 homodimer precursor probed by double electron-electron resonance EPR. Proc. Natl. Acad. Sci. USA.

[104] Tzakos A.G., Grace C.R.R., Lukavsky P.J., Riek R. (2006). NMR techniques for very large proteins and RNAs in solution. Annu. Rev. Biophys. Biomol. Struct..

[105] Tzeng S.R., Pai M.T., Kalodimos C.G. (2012). NMR studies of large protein systems. Methods Mol. Biol..

[106] Frueh D.P., Goodrich A.C., Mishra S.H., Nichols S.R. (2013). NMR methods for structural studies of large monomeric and multimeric proteins. Curr. Opin. Struct. Biol..

[107] Venters R.A., Huang C.C., Farmer B.T., Trolard R., Spicer L.D., Fierke C.A. (1995). High-level 2H/13C/15N labeling of proteins for NMR studies. J. Biomol. NMR.

[108] Weigelt J. (1998). Single scan, sensitivity- and gradient-enhanced TROSY for multidimensional NMR experiments. J. Am. Chem. Soc..

[109] Pervushin K. (2000). Impact of Transverse Relaxation Optimized Spectroscopy (TROSY) on NMR as a technique in structural biology. Q Rev. Biophys.

[110] Tugarinov V., Hwang P.M., Ollerenshaw J.E., Kay L.E. (2003). Cross-correlated relaxation enhanced 1H[bond]13C NMR spectroscopy of methyl groups in very high molecular weight proteins and protein complexes. J. Am. Chem. Soc..

[111] Ollerenshaw J.E., Tugarinov V., Kay L.E. (2003). Methyl TROSY: Explanation and experimental verification. Magn. Reson. Chem..

[112] Rosen M.K., Gardner K.H., Willis R.C., Parris W.E., Pawson T., Kay L.E. (1996). Selective methyl group protonation of perdeuterated proteins. J. Mol. Biol..

[113] Tugarinov V., Kay L.E. (2003). Ile, Leu, and Val methyl assignments of the 723-residue malate synthase G using a new labeling strategy and novel NMR methods. J. Am. Chem. Soc..

[114] Godoy-Ruiz R., Guo C.Y., Tugarinov V. (2010). Alanine Methyl Groups as NMR Probes of Molecular Structure and Dynamics in High-Molecular-Weight Proteins. J. Am. Chem. Soc..

[115] Sheppard D., Sprangers R., Tugarinov V. (2010). Experimental approaches for NMR studies of side-chain dynamics in high-molecular-weight proteins. Prog. Nucl. Magn. Reson. Spectrosc..

[116] Monneau Y.R., Ishida Y., Rossi P., Saio T., Tzeng S.R., Inouye M., Kalodimos C.G. (2016). Exploiting *E. coli* auxotrophs for leucine, valine, and threonine specific methyl labeling of large proteins for NMR applications. J. Biomol. NMR.

[117] Woessner D.E. (1962). Spin Relaxation Processes in a Two-Proton System Undergoing Anisotropic Reorientation. J. Chem. Phys..

[118] Werbelow L.G., Grant D.M., Waugh J.S. (1977). Intramolecular Dipolar Relaxation in Multispin Systems. Advances in Magentic Resonance.

[119] Weiss M.A., Olejniczak E.T. (1990). Are methyl groups relaxation sinks in small proteins?. J. Magn. Reson..

[120] Ishima R., Shibata S., Akasaka K. (1991). General Features of Proton Longitudinal Relaxation in Proteins in Solution. J. Magn. Reson..

[121] Zheng X., Mueller G.A., DeRose E.F., London R.E. (2009). Solution characterization of [methyl-(13)C]methionine HIV-1 reverse transcriptase by NMR spectroscopy. Antivir. Res..

[122] Zheng X., Mueller G.A., Cuneo M.J., Derose E.F., London R.E. (2010). Homodimerization of the p51 subunit of HIV-1 reverse transcriptase. Biochemistry.

[123] Thammaporn R., Yagi-Utsumi M., Yamaguchi T., Boonsri P., Saparpakorn P., Choowongkomon K., Techasakul S., Kato K., Hannongbua S. (2015). NMR characterization of HIV-1 reverse transcriptase binding to various non-nucleoside reverse transcriptase inhibitors with different activities. Sci. Rep..

[124] Seetaha S., Yagi-Utsumi M., Yamaguchi T., Ishii K., Hannongbua S., Choowongkomon K., Kato K. (2016). Application of Site-Specific Spin Labeling for NMR Detecting Inhibitor-Induced Conformational Change of HIV-1 Reverse Transcriptase. ChemMedChem.

[125] Zheng X., Mueller G.A., Kim K., Perera L., DeRose E.F., London R.E. (2017). Identification of drivers for the metamorphic transition of HIV-1 reverse transcriptase. Biochem. J..

[126] Drögemüller J., Strauß M., Schweimer K., Wöhrl B.M., Knauer S.H., Rösch P. (2005). Exploring RNA polymerase regulation by NMR spectroscopy. Sci. Rep..

[127] Gelis I., Bonvin A.M.J.J., Keramisanou D., Koukaki M., Gouridis G., Karamanou S., Economou A., Kalodimos C.G. (2007). Structural basis for signal-sequence recognition by the translocase motor SecA as determined by NMR. Cell.

[128] Slack R.L., Ilina T.V., Xi Z., Giacobbi N.S., Kawai G., Parniak M.A., Sarafianos S.G., Sluis Cremer N., Ishima R. (2019). Conformational Changes in HIV-1 Reverse Transcriptase that Facilitate Its Maturation. Structure.

[129] Mak J., Jiang M., Wainberg M.A., Hammarskjold M.-L., Rekosh D., Kleiman L. (1994). Role of Pr160gag-pol in mediating the selective incorporation of tRNALys into human immunodeficiency virus type 1 particles. J. Virol..

[130] Mak J., Khorchid A., Cao Q., Huang Y., Lowy I., Parniak M.A., Prasad V.R., Wainberg M.A., Kleiman L. (1997). Effects of mutations in Pr160gag-pol upon tRNA(Lys3) and Pr160gag-pol incorporation into HIV-1. J. Mol. Biol..

[131] Khorchid A., Javanbakht H., Wise S., Halwani R., Parniak M.A., Wainberg M.A., Kleiman L. (2000). Sequences within Pr160gag-pol affecting the selective packaging of primer tRNA(Lys3) into HIV-1. J. Mol. Biol..

[132] Sharaf N.G., Gronenborn A.M. (2015). (19)F-modified proteins and (19)F-containing ligands as tools in solution NMR studies of protein interactions. Methods Enzymol..

[133] Sharaf N.G., Ishima R., Gronenborn A.M. (2016). Conformational plasticity of the NNRTI-binding pocket in HIV-1 reverse transcriptase—A fluorine NMR study. Biochemistry.

[134] Mittermaier A., Kay L.E., Forman-Kay J.D. (1999). Analysis of deuterium relaxation-derived methyl axis order parameters and correlation with local structure. J. Biomol. NMR.

[135] Kay L.E., Nicholson L.K., Delaglio F., Bax A., Torchia D.A. (1992). Pulse sequences for removal of the effects of cross correlation between dipolar and chemical-shift anisotropy relaxation mechanisms on the measurement of heteronuclear T1 and T2 values in proteins. J. Magn. Reson..

[136] Takeuchi K., Arthanari H., Shimada I., Wagner G. (2015). Nitrogen detected TROSY at high field yields high resolution and sensitivity for protein NMR. J. Biomol. NMR.

[137] Markus M.A., Dayie K.T., Matsudaira P., Wagner G. (1994). Effect of deuteration on the amide proton relaxation rates in proteins. Heteronuclear NMR experiments on villin 14T. J. Magn. Reson. B.

[138] Ulmer T.S., Campbell I.D., Boyd J. (2004). Amide proton relaxation measurements employing a highly deuterated protein. J. Magn. Reson..

[139] Kalk A., Berendsen H.J.C. (1976). Proton magnetic relaxation and spin diffusion in proteins. J. Magn. Rson..

[140] Lipari G., Szabo A. (1982). Model-Free Approach to the Interpretation of Nuclear Magnetic-Resonance Relaxation in Macromolecules. 1. Theory and Range of Validity. J. Am. Chem. Soc..

[141] Clore G.M., Driscoll P.C., Wingfield P.T., Gronenborn A.M. (1990). Analysis of the backbone dynamics of interleukin-1 beta using two-dimensional inverse detected heteronuclear 15N-1H NMR spectroscopy. Biochemistry.

[142] Xie J., Schultz P.G. (2005). Adding amino acids to the genetic repertoire. Curr. Opin. Chem. Biol..

[143] Peeler J.C., Mehl R.A. (2012). Site-specific incorporation of unnatural amino acids as probes for protein conformational changes. Methods Mol. Biol..

[144] Chiang C.C., Tseng Y.T., Huang K.J., Pan Y.Y., Wang C.T. (2012). Mutations in the HIV-1 reverse transcriptase tryptophan repeat motif affect virion maturation and Gag-Pol packaging. Virology.

